# A Combined Experimental and First-Principles Based Assessment of Finite-Temperature Thermodynamic Properties of Intermetallic Al_3_Sc

**DOI:** 10.3390/ma14081837

**Published:** 2021-04-07

**Authors:** Ankit Gupta, Bengü Tas, Dominique Korbmacher, Biswanath Dutta, Yulia Neitzel, Blazej Grabowski, Tilmann Hickel, Vladimir Esin, Sergiy V. Divinski, Gerhard Wilde, Jörg Neugebauer

**Affiliations:** 1 Max-Planck-Institut für Eisenforschung GmbH, 40237 Düsseldorf, Germany; ankitgupta.tech@gmail.com (A.G.); do.k@gmx.net (D.K.); dutta@mpie.de (B.D.); neugebauer@mpie.de (J.N.); 2 Institute of Materials Physics, University of Münster, 48149 Münster, Germany; b_task01@uni-muenster.de (B.T.); buranova.yulia@gmail.com (Y.N.); divin@uni-muenster.de (S.V.D.); gwilde@uni-muenster.de (G.W.); 3 Institute for Materials Science, University of Stuttgart, Pfaffenwaldring 55, 70569 Stuttgart, Germany; blazej.grabowski@imw.uni-stuttgart.de; 4 Centre des Matériaux (UMR CNRS 7633), MINES ParisTech, PSL University, 91003 Evry, France; vladimir.esin@mines-paristech.fr

**Keywords:** ab initio, Al-Sc alloys, heat capacity, coefficient of thermal expansion, precipitation

## Abstract

We present a first-principles assessment of the finite-temperature thermodynamic properties of the intermetallic Al${}_{3}$Sc phase including the complete spectrum of excitations and compare the theoretical findings with our dilatometric and calorimetric measurements. While significant electronic contributions to the heat capacity and thermal expansion are observed near the melting temperature, anharmonic contributions, and electron–phonon coupling effects are found to be relatively small. On the one hand, these accurate methods are used to demonstrate shortcomings of empirical predictions of phase stabilities such as the Neumann–Kopp rule. On the other hand, their combination with elasticity theory was found to provide an upper limit for the size of Al${}_{3}$Sc nanoprecipitates needed to maintain coherency with the host matrix. The chemo-mechanical coupling being responsible for the coherency loss of strengthening precipitates is revealed by a combination of state-of-the-art simulations and dedicated experiments. These findings can be exploited to fine-tune the microstructure of Al-Sc-based alloys to approach optimum mechanical properties.

## 1. Introduction

Al-Sc alloys form an emerging class of high strength light-weight structural materials and are potential candidates for aerospace and automotive applications. Motivated by their technological relevance, considerable efforts have been made in understanding their thermodynamic and kinetic properties both experimentally and computationally [1,2,3,4,5,6,7,8,9,10,11,12,13,14,15,16,17,18,19,20,21,22]. The microstructure of Al-Sc alloys is characterized by the presence of homogeneously dispersed intermetallic Al${}_{3}$Sc precipitates formed during the Sc solid solution decomposition in Al. These precipitates share the same crystal structure (*L*1${}_{2}$ ordering) [10,23] as the underlying Al matrix, are coherent at nanometer scale due to a small lattice misfit with the matrix, and are responsible for the improved mechanical properties of Al-Sc alloys. For instance, 1 wt.% Sc addition to Al increases its strength by 240 MPa [3,4,5,24]. In addition, Sc is also known to impact grain refinement and to increase the recrystallization temperature significantly, if the Al${}_{3}$Sc precipitates remain small enough. An addition of 0.6 wt.% Sc almost doubles the recrystallization temperature of pure Al [6,7,8].

The emerging technological applications require careful engineering of the precipitation in the Al-based alloys in particular by thermo-mechanical treatments/loads with a focus on the particle/matrix coherency [8]. One promising approach is a designed micro-alloying by carefully selected elements that affect the lattice misfit and limit the particle growth. For example, addition of Zr and/or Ti to Al–Sc-based alloys results in formation of core-shell particles with retarded growth kinetics [25,26].

These concepts require as a first step a complete knowledge of the finite-temperature thermodynamic properties for the binary Al–Sc alloys, for the measured data are surprisingly rare and limited to dilatometric data [12]. We recently published the first calorimetric data [27] of Al${}_{3}$Sc phase up to 400 K together with its detailed ab initio based investigation, explaining the rich feature set observed for T<50 K. On the one hand, calorimetric data of the Al${}_{3}$Sc phase for T>400 K are currently missing. On the other hand, there are discrepancies for the coefficient of thermal expansion (CTE) between the available dilatometric data [12], which yield a constant CTE of 1.6 × 10${}^{-5}$ K${}^{-1}$ between 298–1173 K, and the ab initio predictions, which report both a continuously increasing [22,28] CTE as well as a constant [29] CTE beyond room temperature. Here, the considered finite-temperature excitation effects in the existing ab initio based investigations for Al${}_{3}$Sc still lack the contributions due to the phonon anharmonicity.

While the significance of anharmonic contributions to the thermodynamic properties close to the melting temperatures has recently been highlighted for an extensive set of unary fcc metals [30,31], evaluation of these contributions for alloys is still in its infancy [32,33,34]. The desired precision of < 1 meV/atom in the anharmonic free energies over the whole temperature range makes this task computationally challenging. For this purpose, the recently developed UP-TILD (Upsampled Thermodynamic Integration Using Langevin Dynamics) scheme has proved quite promising [30,31,32,33,34]. Our dilatometric and calorimetric measurements further allow us to perform an integrated assessment of the importance of finite-temperature excitation mechanisms for the thermodynamic properties of Al${}_{3}$Sc.

In this work, we present dilatometric (308–1273 K) and calorimetric (400–773 K) data for the intermetallic Al${}_{3}$Sc phase combined with an ab initio based analysis considering the complete excitation-spectrum including phonon–phonon anharmonicity. We also assess the applicability of standard empirical laws aimed at describing the properties of compounds from the knowledge of elemental species for the example of Al${}_{3}$Sc. Specifically, we evaluate the Vegard’s law for the prediction of lattice parameters and the Neumann–Kopp rule which applies to the heat capacities. The in-depth analysis reveals the limitations of the empirical rules, especially with respect to the ordered Al${}_{3}$Sc alloy. The temperature-dependent critical size for the Al${}_{3}$Sc particles to maintain their coherency with the Al matrix is evaluated and checked against available experimental data.

The paper is organized as follows: the following Section 2 briefly discusses the theoretical background together with the computational and experimental set-up details. In Section 3, we present and discuss the dilatometric and calorimetric data along with the ab initio results. Finally, Section 4 includes a brief summary of the paper with concluding remarks.

## 2. Methodology

In order to perform a first-principles assessment of thermodynamic properties of materials [35], the dependence of the Helmholtz free energy

(1) $$\begin{array}{cc} F(V,T) & =E_{0K}\left(V\right)+F^{\mathrm{el}}\left(V,T\right)+F^{\mathrm{qh}}\left(V,T\right)\\ \; & \;+F^{\mathrm{ph}-\mathrm{ph}}\left(V,T\right)+F^{\mathrm{el}-\mathrm{ph}}\left(V,T\right), \end{array}$$

on volume, *V* and temperature, *T*, is determined within density functional theory (DFT). Here, the relevant, adiabatically decoupled entropy contributions result from electronic excitations (el), quasiharmonic lattice vibrations (qh), an anharmonic phonon–phonon coupling (ph-ph), as well as an electron–phonon coupling (el-ph). The latter contains adiabatic as well as non-adiabatic contributions. The isobaric heat capacity

(2) $$C_{P}=-T{\left(\frac{\partial^{2}F\left(V,T\right)}{\partial T^{2}}\right)}_{V,P}.$$

is often used to benchmark the quality of *ab initio* derived free energies against experiments. The lattice expansion is usually expressed by the coefficient of thermal expansion

(3) $$\alpha \left(T\right)=\frac{1}{a(T)}\frac{\partial a(T)}{\partial T},$$

where a(T) is the lattice constant. In the following, the methods employed for the calculation of the different energy contributions are briefly introduced.

### 2.1. Electronic Contribution

For the electronic contribution $F^{\mathrm{el}}\left(V,T\right)$ the finite-temperature DFT formalism of Mermin [35,36] is used. The dependence on {V,T} is evaluated on an equidistant mesh of 11 volumes and 11 temperatures, rewpectively. Everywhere on this mesh a numerical precision of less than 0.1 meV/atom is ensured. For the *T* parametrization, the electronic density of states is expanded as a third-order polynomial. The *V* parametrization is performed using a second-order polynomial. For further details, we refer to our recent work [27].

### 2.2. Vibrational Contributions

The sum of the terms $F^{\mathrm{qh}}$ and $F^{\mathrm{ph}-\mathrm{ph}}$ in Equation (1) describes the vibrational free energy. The non-interacting, volume-dependent phonons, $F^{\mathrm{qh}}\left(V,T\right)$, are determined within the quasiharmonic approximation [35,37].

The explicit anharmonic contribution to F(V,T) due to phonon–phonon interactions, $F^{\mathrm{ph}-\mathrm{ph}}$, is calculated with the UP-TILD scheme developed by Grabowski et al. [30,32]. Within this scheme, $F^{\mathrm{ph}-\mathrm{ph}}$ at a given {V,T} is calculated as

(4) $$F^{\mathrm{ph}-\mathrm{ph}}=\int_{0}^{1}d\lambda \left[{\langle E_{\mathrm{low}}^{\mathrm{DFT}}-E^{\mathrm{qh}}\rangle }_{\lambda }+{\langle \Delta E\rangle }^{\mathrm{UP}}\right].$$

Here, $E^{\mathrm{DFT}}$ is the full DFT energy, $E^{\mathrm{qh}}$ is the reference DFT potential computed from the Hessian matrices, λ is a coupling parameter and ${\langle \ldots \rangle }_{\lambda }$ represents an average over a canonical ensemble of many uncorrelated atomic configurations that are generated during molecular dynamics simulations with a Langevin thermostat. The values of λ= 0 and 1 correspond to only quasiharmonic atomic forces and full DFT atomic forces, respectively, whereas 0<λ<1 corresponds to a linear coupling between the two. The first ensemble average term ${\langle E_{\mathrm{low}}^{\mathrm{DFT}}-E^{\mathrm{qh}}\rangle }_{\lambda }$ in Equation (4) is obtained using the DFT with relatively low values for the cutoff energy $E_{\mathrm{cut}}$ and the *k*-point mesh. The configuration-independent offset ${\langle \Delta E\rangle }^{\mathrm{UP}}$ between the less an the fully converged energies is treated using free energy perturbation theory. For further details, we refer to Ref. [38].

### 2.3. Electron–Phonon Coupling

The non-adiabatic part of the electron–phonon coupling can be computed using density functional perturbation theory (DFPT). For Al${}_{3}$Sc, our recent investigation showed that the adiabatic influence of electronic temperature on phonons is negligible. The adiabatic part given by the explicit modification of the electronic free energy by the lattice vibrations is computed within the framework of self-consistent field (SCF) finite temperature DFT employing ab initio molecular dynamics (AIMD) simulations. For further methodological details, we refer to References [27] and [39].

### 2.4. Elastic Constants

Since the Al${}_{3}$Sc phase has an fcc-based *L*1${}_{2}$ crystal structure, its complete elasticity/stiffness matrix is determined by only three elastic constants, namely $C_{11}$, $C_{12}$ and $C_{44}$[40]. Using first-principles calculations, the matrix components $C_{ij}$ are obtained with volume conserving orthorhombic and monoclinic strains applied to the crystal. More specifically, the matrix components $C_{11}$, $C_{12}$ and $C_{44}$ are determined by distortions δ [41]. The corresponding energies E(δ) of the crystal structures are then expanded as a function of the small parameter δ around the equilibrium position (E(δ=0)) as

(5) $$\begin{array}{c} E_{\mathrm{ortho}}\left(\delta \right)=E\left(0\right)+C^{\prime }V_{\mathrm{eq}}\delta^{2}+O\left[\delta^{4}\right]+\ldots , \end{array}$$

(6) $$\begin{array}{c} E_{\mathrm{mono}}\left(\delta \right)=E\left(0\right)+\frac{1}{2}C_{44}V_{\mathrm{eq}}\delta^{2}+O\left[\delta^{4}\right]+\ldots , \end{array}$$

where $V_{\mathrm{eq}}$ is the equilibrium volume and $C^{\prime }=C_{11}-C_{12}$ is the shear modulus. The strain energy density *U* is obtained as $U\left(\delta \right)=[\left(E\left(\delta \right)-E\left(0\right)\right)/V_{\mathrm{eq}}]$, ignoring terms of order $O[\delta^{4}]$, and the second derivative yields $\frac{\partial^{2}U_{\mathrm{ortho}}}{\partial \delta^{2}}=2\left(C_{11}-C_{12}\right)$ and $\frac{\partial^{2}U_{\mathrm{mono}}}{\partial \delta^{2}}=C_{44}$. For cubic crystals, $C_{11}$ and $C_{12}$ are related to the bulk modulus as $B_{M}=\frac{1}{3}\left(C_{11}+2C_{12}\right)$, where $B_{M}$ is obtained by fitting energy-volume curves to the Murnaghan equation-of-state [42].

### 2.5. Computational Details

In order to ensure comparability, the computational details of the present work are to a large extent identical to those in our previous study on low-temperature features in Al and Al${}_{3}$Sc [27] and are, therefore, only summarized here. For further computational details we refer to [43]. The DFT calculations are performed using the Vienna Ab Initio Simulation Package (vasp) [44,45] and the projector-augmented wave (PAW) method [46]. As exchange-correlation functional the generalized gradient approximation (GGA) within the parameterization of Perdew, Burke and Ernzerhof (PBE) [47] is used.

The phonon calculations are performed using a 4×4×4 fcc supercell and the small displacement method [48,49]. A displacement value of 0.02 Bohr radius (≈0.01 Å) ensures a linear dependence of the forces on the displacement. The plane-wave cutoff energy *E*${}_{\mathrm{cut}}$ = 400 eV and a 6×6×6 *k*-mesh for the sampling of the Brillouin zone (BZ) using a Monkhorst-Pack scheme [50] are employed. The criterion for energy convergence is $10^{-7}$ eV. The Methfessel–Paxton scheme [51] is employed with a width of 0.15 eV.

The temperature dependence of the electronic free energy with 11 temperature steps between 1–1590 K is modelled by a Fermi smearing with a width ranging from $8.6\times 10^{-5}$ eV to 0.137 eV. For these calculations, a unit cell with a k-mesh of 20×20×20 grid points and a plane-wave energy cutoff of 300 eV are used in order to ensure a precision of $10^{-3}$ meV/atom.

The calculations of the electron–phonon coupling are performed with the ABINIT code [52,53] using density functional perturbation theory (DFPT) [54] and PAW potentials (different than those in VASP). In this case, the LDA exchange–correlation furnctional parametrized by Perdew and Wang [55] was used, since the current ABINIT implementation does not support DFPT for GGA. A cutoff energy of 20 Ha (544 eV) and a *k*-point mesh of 28×28×28 yield a precision of λ on the order of $10^{-3}$. A convergence criterion for the self-consistent field cycle on the wave function squared residual of $10^{-14}$ Ha (2.72×10${}^{-13}$ eV) is chosen. For the calculations of Al${}_{3}$Sc the equilibrium lattice parameter of 4.032 Å (7.62 Bohr) has been chosen, which is almost identical to the LDA lattice constant obtained with VASP (4.033 Å).

### 2.6. Experimental Details

Dilatometric and calorimetric experiments were performed on pure Al (99.999 wt.%) and an Al${}_{3}$Sc alloy. The Al${}_{3}$Sc ingot was prepared by the research group of M. Rettenmayr (Otto Schott Institute of Materials Research, Friedrich-Schiller-University Jena, Germany) by induction melting from pure Al (99.999 wt.%) and Sc (99.99 wt.%) and casting into a copper mold under vacuum conditions. The ingot was remelted several times and homogenized at 1273 K for 17 h. The phase composition was checked by X-ray diffraction using a Siemens D5000 device and locally by energy-dispersive X-ray analysis with an FEI Nova NanoSEM 230 scanning electron microscope (for further details see Ref. [27]).

Nanometer-scale Al${}_{3}$Sc-based precipitates were investigated in a Al-based commercial AA5024 alloy as a case study. The chemical composition corresponded to an Al-4.6Mg-0.35Mn-0.2Sc-0.09Zr-0.02Ti (in wt.%) alloy. The production and preparation steps are summarized in Ref. [27]. In the present study, the main focus was on the Al${}_{3}$Sc-based precipitates. High-resolution transmission electron microscopy (HR-TEM) including high-annular dark field scanning TEM (HAADF-STEM) imaging was performed on a Zeiss Libra 200FE with a camera length of 720 mm, a collection angle of 65 mrad outwards and a probe size of 1.2 nm. The strain fields in the HR-TEM images were quantified using the geometric phase analysis (GPA) method [56]. The local displacements of atom columns with respect to a reference (taken as non-distorted) lattice were characterized by in-plane shear, $\epsilon_{xy}$, and rotational, $\omega_{xy}$, strain tensor components, calculated using a commercial version, GPA Phase 2.0 (HREM Research), as a plug-in for DigitalMicrograph (Gatan).

#### 2.6.1. Low Temperature Heat Capacity Measurements

The low temperature heat capacities of pure Al and the Al${}_{3}$Sc alloy were measured using a Physical Properties Measurement System (PPMS), Quantum Design. During measurements, a heat pulse was supplied to the sample and its response (temperature change) was recorded. The measurements were performed in the temperature range of 1.9 to 400 K under high-vacuum conditions.

Each measurement run includes the heat capacity measurements of the solely sample holder (addenda) and the sample itself. At a given temperature, an individual measurement step consists of heating and cooling cycles. During an applied heating step, the temperature change of the sample material is followed for a predefined time until equilibration, and subsequently the sample is allowed to cool for the same time. The heat capacity is determined using a two-tau model [57] which takes into account a non-ideality of the thermal contact between the sample and the heating platform. Each such measurement at the given temperature is repeated three times and an average value is determined.

The determined heat capacity values include the effects from the sample, grease, and the platform. The addenda measurement yields the heat capacity of the grease (accordingly, the same amount of grease is used in both sets) plus platform set-up and the final heat capacity of the sample is determined by subtraction of the addenda measurement from the total heat capacity value.

#### 2.6.2. Dilatometric Measurements

A Linseis (L70/2171) dilatometer, which allows determination of the length changes with an absolute accuracy of about 100 nm was employed to measure the length changes of pure Al during heating in the temperature interval from 300 K to 773 K. Specimens were cut as cylinders of the length of 20 mm and 3 mm in diameter. The flat surfaces were carefully polished and mounted vertically in the measurement set-up. Three runs were performed and the thermal elongation, ΔL, was proven to be identical for the second and third runs. The values of a relative elongation, $\Delta L/L_{0}$, where $L_{0}$ is the initial sample length at room temperature, determined in the second run are used.

Temperature-induced length changes of Al${}_{3}$Sc were performed on rectangular 2×2×14.9 mm${}^{3}$ samples using a Netzch DIL 402 CD horizontal dilatometer in the temperature interval from 300 K to 1273 K and heating/cooling rate of 2 K min${}^{-1}$. An absolute accuracy in the determination of the length changes was about 1 nm. In total, four measurement runs were performed including heating and cooling to/from 1173 K followed by heating/cooling runs to/from 1273 K. Each experiment with the sample was preceded by a correction test using a cylindrical Al${}_{2}$O${}_{3}$ standard of 25 mm length and 6 mm diameter. Such a correction was required to be able to subtract the “apparatus response” to the heating/cooling. Before each experiment, the dilatometer furnace was purged three times using Ar and the experiments were also carried out in static Ar atmosphere to prevent sample oxidation. However, very slight surface oxidation was observed that should not disturb the experimental results. All obtained $\Delta L/L_{0}$ data as a function of temperature were perfectly superposed indicating an excellent reproducibility of experimental results. It is worth noting that the imposed heating rate of 2 K min${}^{-1}$ was not respected on heating before the temperature has reached the value of 420 K: faster and slower not constant heating rates were observed between 300 and 420 K. Therefore, the results obtained in this temperature range should be analyzed with particular attention.

The temperature dependent linear thermal expansion coefficients at a given temperature *T* were determined via cubic-spline fitting of the measured data points within the interval of ±30 K with respect to the temperature *T* in question. The value of ±30 K was found to provide a good compromise between the requirement to filter the high-frequency irregularities and a reliable representation of potential nonlinear features of the thermal expansion.

## 3. Results and Discussion

### 3.1. Ground State Properties

The calculated equilibrium parameters of Al${}_{3}$Sc are listed in Table 1 along with the experimental data. The ground state lattice parameter value is 4.103 Å. The computed value of 4.124 Å at ambient temperature agrees well with the measured value [12,23] of 4.103 Å. The electronic and anharmonic contributions to the lattice parameter at room temperature are found to be negligible. The computed elastic constants of Al${}_{3}$Sc are laying within ±10% of the measured values [58,59] as well as previous calculations [10,14,60,61,62]. The Born stability criterion for the mechanical stability of cubic structures is fulfilled for Al${}_{3}$Sc: $C_{11}>\left|C_{12}\right|$, $C_{44}>0$ and $C_{11}+2C_{12}>0$.

### 3.2. Heat Capacity

In order to assess the predictive capability of the ab initio based thermodynamics outlined in the previous chapter, calorimetric measurements are often used as a benchmark. Being the second derivative of F(V,T) with respect to temperature, they are very sensitive to the treatment of finite-temperature excitations. In addition, thermodynamic databases, for instance in CALPHAD, heavily exploit heat capacity data. Our recent [27] first principles analysis of calorimetric data for Al and Al${}_{3}$Sc provided us with a fundamental understanding of the intriguing features observed in the low-temperature regime. Electronic excitations and a non-adiabatic electron–phonon coupling are responsible for a very good agreement with measurements up to 400 K. Existing theoretical predictions for the heat capacity of Al${}_{3}$Sc beyond 400 K (up to 1000 K) are either based on the harmonic or quasiharmonic approximation [28,63] and neglect the volume dependence of the electronic free energy [29], phonon anharmonicity, and the explicit electron–phonon coupling.

**Figure 1:**
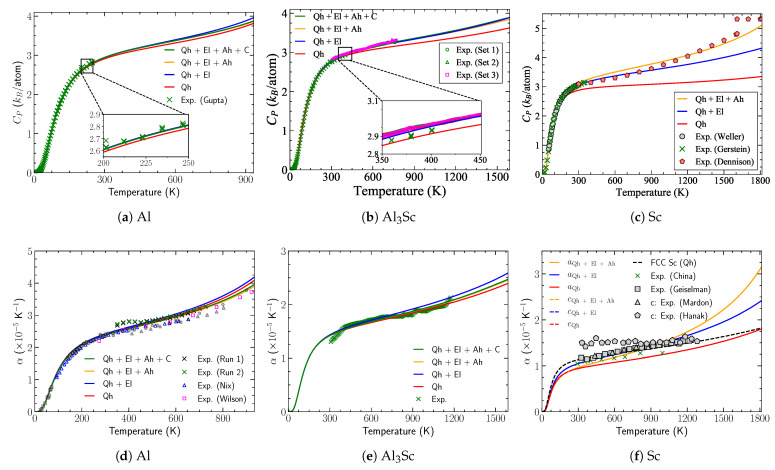
Calculated and measured (**a**)–(**c**) isobaric heat capacity and (**d**)–(**f**) coefficient of thermal expansion of fcc Al, *L*1${}_{2}$ Al${}_{3}$Sc and hcp Sc including quasiharmonic (Qh), electronic (El), and anharmonic (Ah) excitations as well as el-ph coupling (**c**). In addition, the coefficient of thermal expansion of Sc in the fcc phase is given in (**f**). The literature values of CTE (blue triangles, magenta squares, and all gray symbols) for Al in (**d**) are taken from Refs. [64], [65] and [66]. Experimental calorimetric data are included as green crosses [27] in (**a**) and (**b**). Sc thermal expansion data are included as triangles [67], pentagons [68], hexagons [69]. The measured data for Sc heat capacity are included as crosses [70], pentagons [71], and circles [72]. The figures have been adapted from [43].

In Figure 1b, we present the calculated $C_{P}$ of Al${}_{3}$Sc from 0–1590 K including all relevant entropy contributions together with the first calorimetric data for 400–773 K and our previous measurements [27] up to 400 K. The electronic contribution starts becoming relevant above room temperature and is about 0.3 $k_{B}/\mathrm{atom}$ at the melting point, $T_{m}$. In fact, it can be seen that the quasiharmonic approximation (red curve) fails to reproduce the measurements above 300 K. By contrast, the anharmonic contribution is found to be small and negative around −0.04 $k_{B}/\mathrm{atom}$ (≈1% of Qh+El contributions) and largely compensated by the electron–phonon coupling.

The pure element Sc shows a substantial electronic contribution to $C_{P}$, ≈ 1 $k_{B}/\mathrm{atom}$ at $T_{m}$. In Al, the electronic contribution is 0.15 $k_{B}/\mathrm{atom}$, i.e., almost an order of magnitude smaller, and there are small corrections from anharmonicity and electron–phonon coupling. Such a trend can already be observed in the electronic contributions to the free energy for Al (−4.5 meV/atom), Al${}_{3}$Sc (−13.6 meV/atom), and hcp Sc (−85 meV/atom) at the melting point. It is due to the electronic DOS, which shows for Sc a sharp peak at the Fermi level with 2.5 states/eV-atom, while Al and Al${}_{3}$Sc have a reduced DOS at the Fermi level, approximately 0.4 and 0.56 states/eV-atom. More generally, early series of 3d, 4d and 5d transition metals [39] tend to have a higher DOS at the Fermi level than transition elements like Cu, Ag, Au, Zn, Cd and Hg that are on the right-hand side of the periodic table. The high energetic stability of the Al${}_{3}$Sc intermetallic compound is also related to its reduced DOS at the Fermi level.

Motivated by the electronic contribution, the question arises regarding whether information about the compounds can be deduced from the thermodynamics of the elemental species. One example for such an empirical approximation is the Neumann−Kopp rule (NKR), stating that the molar heat capacity of a compound A${}_{1-c}$B${}_{c}$ can be obtained by a linear-weighted combination of the molar heat capacities of its constituents as [73],

(7) $$C_{P}\left(A_{1-c}B_{c}\right)=\left(1-c\right)C_{P}\left(A\right)+cC_{P}\left(B\right).$$

More generally, for material with *N* components, the NKR is represented as $C_{P}=\Sigma_{i}^{N}f_{i}^{m}C_{P}\left(i\right)$, $f_{i}^{m}$ being the molar fraction of constituent *i*. The heat capacities of pure elements have to be taken in per mole units (J mol${}^{-1}$ K${}^{-1}$). If better calorimetric data are not available, the NKR is nowadays a standard method for constructing phase diagrams (e.g., within the CALPHAD approach) of multicomponent alloys, despite the fact that changes in the electronic structure due to the formation of compounds from the constituents are not taken into account. Besides its usual application to estimate heat capacities, equations similar to Equation (7) have also been applied to other thermodynamic quantities including the enthalpies of fusion [74], Gibbs free energy [75] and entropies of formations [76] of various compounds.

**Figure 2:**
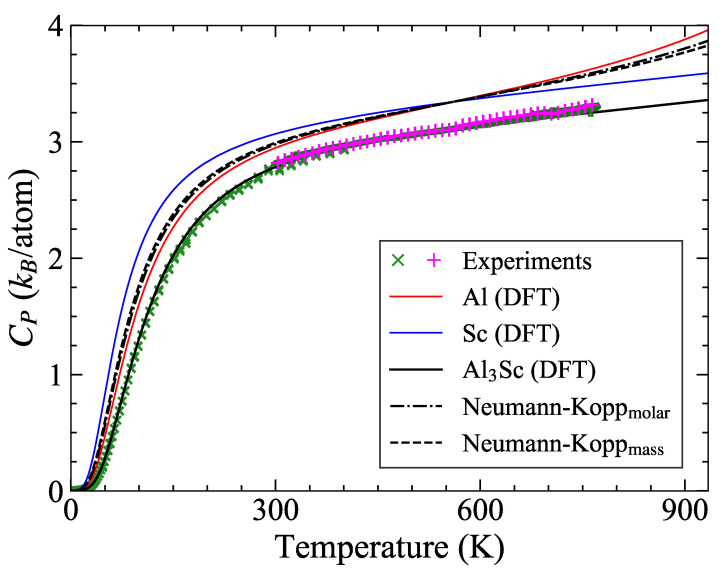
Isobaric heat capacity of Al${}_{3}$Sc as obtained by DFT calculations (solid lines), which include the quasiharmonic and electronic contributions, and by using the molar and mass weighted Neumann-Kopp rule (Equation (7)) for Al${}_{3}$Sc using (dashed lines). Calorimetric data are given by green and magenta symbols. The figure has been adapted from [43].

Figure 2 shows the $C_{P}$ of Al${}_{3}$Sc estimated with the NKR using ab initio inputs for fcc Al and hcp Sc. Here, we limit ourselves to the quasiharmonic and electronic contributions to the heat capacity because Figure 1 indicates qualitative differences between the anharmonicity of fcc Al and that of hcp Sc. By construction, the predictions of Equation (7) (black dashed-dotted curve) will always fall between the end-member heat capacities, while this is not the case for the experiments and the explicit ab initio calculations for Al${}_{3}$Sc. Among the involved phases, Al has the lowest melting point ($T_{m}=934$ K) providing an upper limit for the plot. Additional consideration of liquid-Al $C_{P}$ for T>934 K in Equation (7) even yields an abnormal $C_{P}$ behavior [77,78,79]. We also computed the mass-weighted average and found similar results (black dashed curve) as for the molar-weighted case.

In many cases [80,81,82,83,84,85] the predictive power of the NKR turned out to be surprisingly high, but there have also been several reports about significant deviations of calorimetric data over extended temperature ranges and even at low temperatures[82,86,87]. Grimvall [73] stated certain conditions for the inapplicability of NKR, which included the presence of anharmonic effects and significant non-vibrational contributions in either one or both of the elemental constituents. In the present case, however, this cannot explain the deviations already at room temperature.

The NKR was found to be applicable in several compounds [88] whose molar volumes are nearly equal to the weighted-sum of the elemental molar volumes. The ab initio computed molar volume of Al${}_{3}$Sc is $V_{m}\left({\mathrm{Al}}_{3}\mathrm{Sc}\right)=10.4\times 10^{-6}$ m${}^{3}$ mol${}^{-1}$, in agreement with the experimental value [16] of $10.35\times 10^{-6}$ m${}^{3}$ mol${}^{-1}$. This is lower than the stoichimetric average $11.15\times 10^{-6}$ m${}^{3}$ mol${}^{-1}$ of the ab initio values for $V_{m}\left(\mathrm{Al}\right)=9.92\times 10^{-6}$ and $V_{m}\left(\mathrm{Sc}\right)=14.82\times 10^{-6}$ m${}^{3}$ mol${}^{-1}$ and can therefore explain the underestimation of the heat capacity. Such a condition for assessing the applicability of NKR, however, has been found contradictory for several compounds [82,89]. Furthermore, it cannot been used as a basis for a revised NKR, since the molar volume of the alloy is still between those of the end members.

The situation is more promising for the bulk modulus, since the computed values (at 0 K) of Sc, Al and Al${}_{3}$Sc are 58, 77.3, and 89.6 GPa, respectively. This can be used for an extrapolation scheme, since a lower heat capacity at a given absolute temperature correlates to a higher Debye temperature, and a higher Debye temperature to a higher bulk modulus. Accordingly, we have tried the following approach: The low temperature $C_{V}$ (at low temperatures, $C_{P}$ and $C_{V}$ are similar) data of unaries is fitted to the Debye expression of heat capacity and the corresponding Debye temperatures are obtained, $\theta_{D}^{\mathrm{Al}}=382.3$ K and $\theta_{D}^{\mathrm{Sc}}=297.2$ K. The bulk modulus (0 K) and $\theta_{D}$ of the unaries are fit to a linear relationship. Next, the computed bulk modulus of Al${}_{3}$Sc is used to predict the Debye temperature ($\theta_{D}^{{\mathrm{Al}}_{3}\mathrm{Sc}}=442.9$ K) from a linear fit. The latter is employed in the Debye expression to get the corresponding heat capacity. This approach yields a $C_{V}$ up to the room temperature that agrees very well with the DFT results for Al${}_{3}$Sc. We tested this approach for several other binary compounds, such as Mg${}_{2}$Si, MgNi${}_{2}$, TaCr${}_{2}$, NbCr${}_{2}$ and also found a good agreement below room temperatures with the existing computed/measured data. One inherent limitation of this suggestion is that the Debye expression has an asymptotic limit at 3 $k_{B}/$atom, whereas $C_{P}$ goes beyond this value. Nevertheless, we believe that there is a scope of applying and extending this methodology within a high-throughput framework to screen a large database of compounds, taking finite-temperature contributions into account. It will be a part of our future studies.

### 3.3. Lattice Expansion

#### 3.3.1. Verifying Vegard’s Law for the Sc Solid Solution

Similarly to the heat capacity, the lattice parameter of an alloy is also supposed to vary linearly as a function of the solute concentration *c*. The linear combination $a=\left(1-c\right)a_{A}+ca_{B}$ of the lattice parameters $a_{A}$ and $a_{B}$ for the end members is called Vegard’s law. An equivalent empirical rule that assumes a linear relation between the volume and the concentration is known as Retger’s law. Due to the nonlinear relation between lattice parameter and volume, both laws cannot be fulfilled simultaneously [90]. This is only possible for the coefficients of linear and volume thermal expansion, since $\alpha_{\mathrm{linear}}=\frac{1}{3}\alpha_{\mathrm{volume}}$.

**Figure 3:**
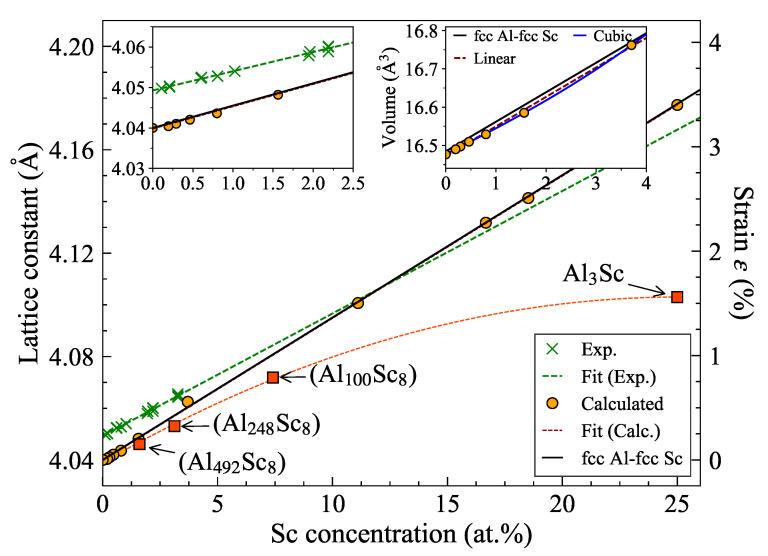
Variation of lattice parameter of substitutional Sc solid solution with Sc concentration in fcc Al. The symbols represents the calculated (orange circles: solid solution, red squares: *L*1${}_{2}$ ordering of Sc atoms) and the measured (green crosses) data points [91]. The dashed lines are fits to the respective data points. The black (solid) line represents the linear interpolation between fcc Al and fcc Sc lattice parameters (Vegard’s law). The left inset provides a zoom into low concentrations. The right inset shows the interpolation for the concentration dependence of the volume (per atom) of the substitutional solid solution (black line: Retger’s law). The figure has been taken from [43].

Figure 3 shows the variation of the Al-Sc solid solution lattice parameter with Sc concentration. On the one hand, $a(c_{\mathrm{Sc}})$ is found to vary linearly with the solute concentration yielding a normalized lattice parameter gradient w.r.t. solute concentration, $\frac{1}{a}\left(\frac{da}{dc}\right)\approx 0.136$ at.%${}^{-1}$. The linear fit also agrees closely with the linear interpolation between the fcc-Al (4.04 Å) and fcc-Sc (4.59 Å) lattice parameters (Vegard’s law). On the other hand, deviations from the linear interpolation of the solid solution volume (Retger’s law) are observed between the fcc-Al and fcc-Sc volume (right inset in Figure 3).

The low solubility of Sc in Al (≈ 10${}^{-10}$ at.% at room temperature and 0.26 at.% at 934 K) makes it difficult to determine the relative change of lattice parameter experimentally. However, quenching the alloy enhances the Sc solubility up to 3.28 at.% which resulted in the experimental value [91] of $\frac{1}{a}\left(\frac{da}{dc}\right)=$ 0.122 ± 0.011 at.%${}^{-1}$. In general, deviations from Vegard’s law have been shown to increase with increasing lattice mismatch between the constituent elements even for a statistically random distribution of solute atoms [90]. Since the elemental Al and Sc have different crystal structures, fcc and hcp respectively, a direct application of this rule is not possible.

The structures for which $a(c_{\mathrm{Sc}})$ is found to vary linearly in Figure 3 correspond to a solid solution in which the Sc atoms were randomly distributed in the Al matrix (SQS configurations). To see how the lattice parameter varies due to the Sc ordering on sub-lattice sites corresponding to *L*1${}_{2}$ crystal structure, we considered several structures (Al${}_{N}$Sc${}_{8}$, red squares in Figure 3) containing eight Sc atoms occupying the corner sites (second nearest-neighbor) of an fcc lattice forming a complete *L*1${}_{2}$ unit cell embedded in an Al matrix. The ordered Al${}_{3}$Sc structure is the limiting case of this arrangement where all the corner sublattices will be occupied by Sc alone. For all Al${}_{N}$Sc${}_{8}$ structures with *L*1${}_{2}$ ordering of Sc atoms, the relaxed lattice parameters show deviation from the linear behavior exhibited by the Sc solid solution, lying below the linear extrapolation. This indicates the inapplicability of Vegard’s law to estimate the lattice parameter of ordered compounds. For the stoichiometric Al${}_{3}$Sc phase of interest, the random solid solution with 25% Sc (represented by an SQS) agrees perfectly with the Vegard’s law. These observations suggest that the deviation of Al${}_{3}$Sc lattice parameter from Vegard’s law and the nonlinearity of $a(c_{\mathrm{Sc}})$ in Figure 3 are primarily caused by chemical ordering within the Al matrix. This also applies to the structures with Sc sublattice ordering for lower Sc concentrations (red squares in Figure 3).

The observed deviation (nonlinearities) of the lattice parameter of structures with local ordering on the Sc sublattice as opposed to the predictions of Vegard’s law for solid solutions in the low solute concentration regime is related to the strong hybridization between *d* electron states of Sc and the *p* states of Al [92] in the *L*1${}_{2}$ arrangement (first nearest-neighbor sites). The latter is also responsible for the high stiffness of Al-Sc bonds in Al${}_{3}$Sc and the stability of these precipitates. While negative deviations are observed for Al-Sc, positive deviations have also been reported for several systems [93,94]. Several other known reasons assigned to the deviations from Vegard’s law include the effect of intermetallic compounds, strain energy, differences in compressibilities, electronegativities, volumes, and the atomic-sizes of the constituents, and short-range ordering in the alloy [94,95].

#### 3.3.2. Coefficient of Thermal Expansion

The computed coefficients of thermal expansion (CTE) for pure Al and Sc are plotted in Figure 1d,f. The ab initio results for Al agree well with our dilatometric data and the literature values over the whole temperature range up to the melting point ($T_{m}=934$ K). As compared to Sc, the electronic contribution of Al is small, even close to the melting point. Including the phonon anharmonicity and the electron–phonon coupling yield a reduction in the thermal expansion, shifting curves below the quasiharmonic approximation. Although the combined impact of the entropy contributions beyond quasiharmonic phonons is for Al around 3% at $T_{m}$, a close look near $T_{m}$ shows the importance of these effects in capturing the correct behavior of experiments. Such fine accuracy becomes important in predictive modeling of alloys and their precipitates.

Due to the limited availability of measured CTE for Sc, a direct comparison of ab initio results with experiments can only be established up to 1300 K (Figure 1f). This temperature regime already clearly indicates the importance of the electronic contribution to achieve a good agreement, whereas anharmonicity yields a substantial contribution above 1300 K. Our calculations show similar values of the CTE for both *a* and *c* lattice parameters. This indicates that hcp Sc is isotropic, which is consistent with anisotropy factors close to the one seen for the elastic behavior.

In the case of the Al${}_{3}$Sc phase, Figure 1e demonstrates a good agreement between the calculated and measured CTE over the whole temperature range of measurements. The CTE being the normalized slope of linear thermal expansion is a sensitive quantity and hence small fluctuations in the measurements of the latter will be reflected as a significant difference in the CTE. This is the reason for the observed fluctuating trend in the measured data points (green crosses).

We compare our calculations and dilatometric data against the only existing measurements by Harada et al. [12] who reported a constant CTE value of 1.6 × 10${}^{-5}$ K${}^{-1}$ between 298–1173 K and 1.77 × 10${}^{-5}$ K${}^{-1}$ between 1173–1273 K. By contrast, our measurements show an increasing trend with CTE varying between 1.55–2.1 × 10${}^{-5}$ K${}^{-1}$ in the temperature range of 400–1173 K. The calculated CTE including electronic and anharmonic contributions at room temperature is 1.41 × 10${}^{-5}$ K${}^{-1}$.

At the melting temperature (1590 K), the contribution of electronic excitations to the CTE of Al${}_{3}$Sc is significant, approximately 8% of the contribution due to the lattice vibrations. The explicit anharmonic contribution, however, is negative, roughly about 5% and is found to partly cancel out the electronic contribution. The quantitative and qualitative impact of anharmonicity on the CTE in Al${}_{3}$Sc is remarkable, given the opposite behavior in Sc. In the latter case, the anharmonic contribution is positive and approx. 15% at the melting point. This indicates that the explicit anharmonicity of the pure elements cannot be used to estimate the anharmonic contribution of the binary compound. The CTE contribution caused by the impact of lattice vibrations on the electronic free energy turns out to be an order of magnitude smaller in Al${}_{3}$Sc than the individual effects.

### 3.4. Critical Particle Size for Coherency Loss

**Figure 4:**
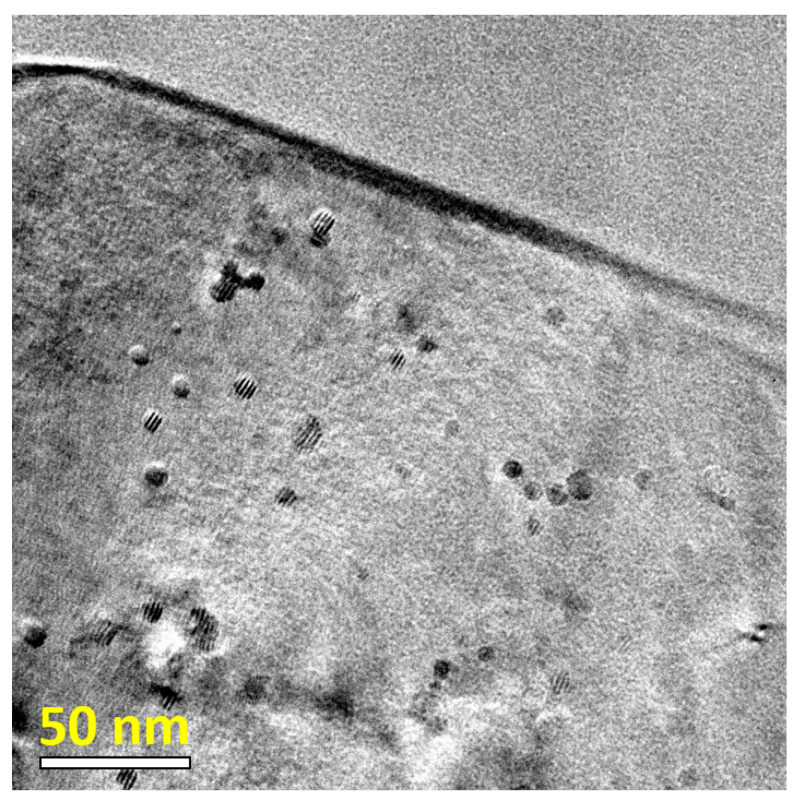
A bright-field TEM image of Al-Sc-based alloy with numerous nanoscale Al${}_{3}$Sc-based precipitates.

The distribution, chemistry, and evolution of Al${}_{3}$Sc-based particles in Al matrix were extensively analyzed in Ref. [27]. Both almost pure Al${}_{3}$Sc and Al${}_{3}$(Sc,Zr) precipitates were observed, see Figure 4, with the latter being often found as core–shell structures with Al${}_{3}$Sc-dominant cores and Al${}_{3}$Zr-dominant shells [27]. In principle, the clearly observed Moiré contrast could be used for a statistical quantification of the interfacial misfit strain around the precipitates [96].

**Figure 5:**
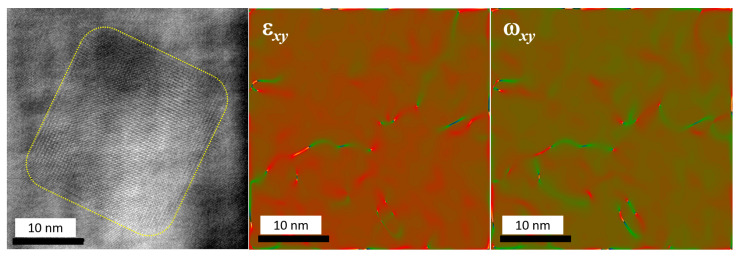
HR-TEM of Al${}_{3}$Sc-based precipitate (left panel) with the results of a geometric phase analysis showing in-plain shear, $\epsilon_{xy}$, (central panel) and rotational $\omega_{xy}$, (right panel) strain components. The precipitate is schematically outlined (left panel).

In the present study, we were focused on the coherency of the particle/matrix interfaces for Al${}_{3}$Sc particles using HR-TEM. In Figure 5, left panel, an about 30 nm large particle is shown as an example. Due to a cube-on-cube misorientation relationship, the crystalline lattices of the particle and the matrix could be resolved using the same zone axis and an almost perfect coherency is seen. The geometric phase analysis, Figure 5, central and right panels, manifests a relatively low density of dislocations, both in the matrix and in the particle, suggesting semi-coherency of particle and matrix.

**Figure 6:**
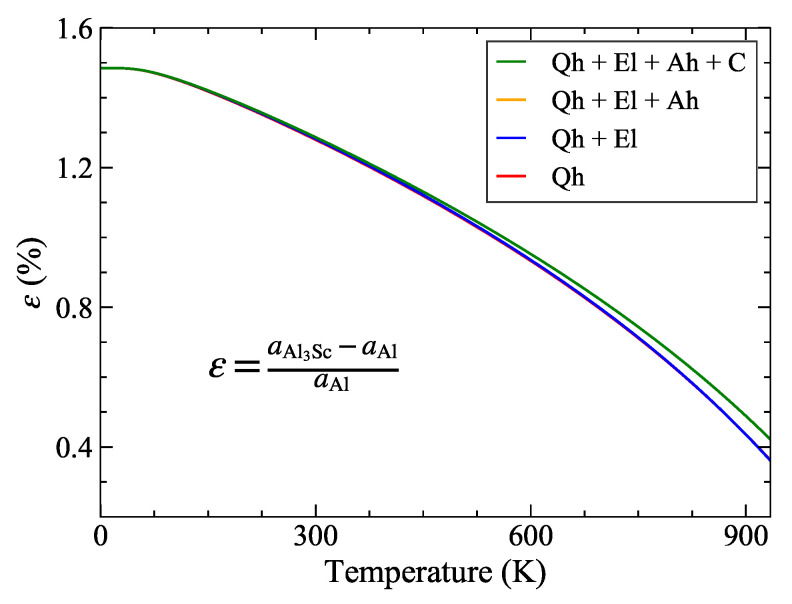
Variation of Al/Al${}_{3}$Sc lattice misfit with temperature computed using the stated equation. The quasiharmonic (Qh), electronic (El) and anharmonic (Ah) and electron-phonon coupling (C) contributions are taken into account in the DFT calculations. The red (orange) lines are hidden by the blue (green) lines. The figure has been taken from [43].

In order to investigate this coherency, we need to compare the thermal expansion behavior of the matrix and the precipitate phase in Al-Sc system. At 0 K, the lattice misfit between Al and Al${}_{3}$Sc is around 1.7% and the equilibrium lattice parameter of Al${}_{3}$Sc is slightly larger than that of Al. The coefficient of thermal expansion of Al (Figure 1d) increases more rapidly with temperature as compared to that of Al${}_{3}$Sc (Figure 1e), which eventually yields a reduction in the lattice mismatch with temperature. This difference in their thermal expansion behaviors also results in a temperature dependence of the lattice misfit which determines the strain at the interface between the two. The lattice misfit between Al and Al${}_{3}$Sc (Figure 6) shows a strong temperature dependence varying significantly from 1.7% at room temperature to 0.4% at 934 K (melting point of Al). The resulting small coherency strain at the Al/Al${}_{3}$Sc interface is one of the key reasons for the high stability of these precipitates even at high temperatures.

The strain due to the lattice mismatch ε at the Al/Al${}_{3}$Sc interface grows with the precipitate size, resulting in its coherency loss from the matrix beyond a certain size. This critical size of the particle, $r_{\mathrm{crit}}$, can be determined from elasticity theory. In general, a precipitate of radius *r* embedded in a matrix material yields an elastic strain energy $E_{\mathrm{str}}$. On the one hand, a loss in coherency yields a decrease in this elastic strain energy. On the other hand, the resulting interfacial dislocation at the precipitate-matrix interface yields an increase in the energy of the interface $E_{\mathrm{int}}$. Therefore, the matrix-precipitate system does not change its energy. In an earlier work [97], it was assumed that elastic strains do not influence the chemical contribution to the interfacial energy and therefore the changes in the misfit strains accompanying the coherency loss only modify the elastic part. Accordingly, $r_{\mathrm{crit}}$ is determined from the energy conservation by equating the two parts [22,98]:

(8) $$\underset{E_{\mathrm{str}}}{\underset{︸}{8\pi r_{\mathrm{crit}}^{3}G\varepsilon^{2}\frac{1+\nu }{3(1-\nu )}}}=\underset{E_{\mathrm{int}}}{\underset{︸}{4\pi r_{\mathrm{crit}}^{2}\sigma_{\mathrm{dis}}}}.$$

Here, *G* is the shear modulus and ν is the Poisson’s ratio of the matrix, and $\sigma_{\mathrm{dis}}$ is the energy (per unit area) of the dislocation network at the interface defined as

(9) $$\begin{array}{cc} \sigma_{\mathrm{dis}} & =\frac{G|\vec{b}\left(T\right)|}{2\pi^{2}}\\ \; & \times \left[\underset{X(T)}{\underset{︸}{1+\beta -\sqrt{\left(1+\beta^{2}\right)}-\beta \ln\left\{2\beta \sqrt{\left(1+\beta^{2}\right)}-2\beta^{2}\right\}}}\right] \end{array}$$

It depends on the Burgers vector $\vec{b}$ of the Al matrix and the decrease in the lattice misfit $\varepsilon^{\prime }\left(T\right)$ caused by the introduction of interfacial dislocations expressed entering $\beta =\pi \varepsilon^{\prime }\left(T\right)/\left(1-\nu \right)$. Solving Equation (8) results in the following expression of the critical radius $r_{\mathrm{crit}}$:

(10) $$r_{\mathrm{crit}}\left(T\right)=\frac{b(T)}{\varepsilon^{2}\left(T\right)}\frac{3(1-\nu )}{4\pi^{2}\left(1+\nu \right)}X\left(T\right)\;$$

where X(T) was defined in Equation (9). The temperature dependence of $r_{\mathrm{crit}}$ comes from the temperature dependent lattice misfit and the Burgers vector. For fcc Al matrix, $|\vec{b}|=b_{110}=\frac{a_{\mathrm{Al}}\sqrt{2}}{2}$ where $a_{\mathrm{Al}}$ is the lattice parameter of Al. The typical value [99] of ν for Al is around 0.34.

**Figure 7:**
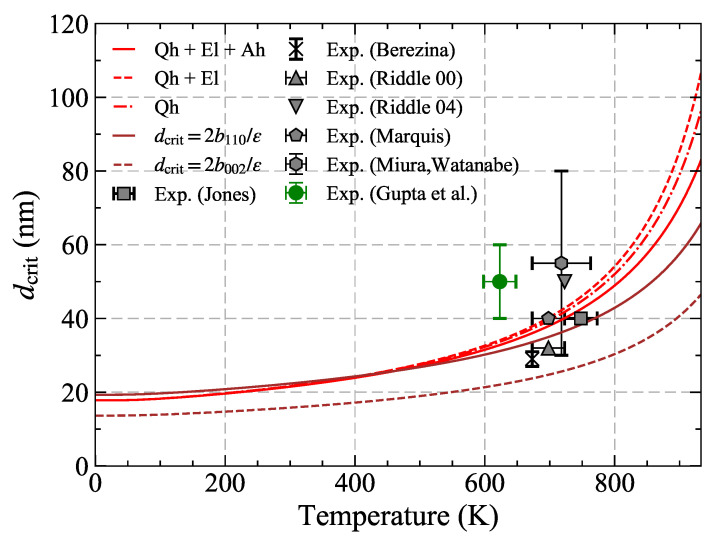
Variation of critical particle diameter of Al${}_{3}$Sc particles with temperature. The red dashed-dotted/dashed/solid lines correspond to the calculated values as per Equation (10) including quasiharmonic (Qh), additional electronic (Qh+El), and additional anharmonic (Qh+El+Ah) contributions to ε(T) and $\vec{b}\left(T\right)$. The symbols hexagons [98,102], up-triangles [103], square [104], crosses [105], down-triangles [106], pentagons [16] represent average experimental values for the coherency loss, whereas the variation in terms of temperature and size is given by error bars. The green symbol corresponds to our recent measurements [101]. The solid and dashed brown lines correspond to the calculation of critical diameters within a pure geometric consideration for two choices of the Burgers vector. The figure has been taken from [43].

Previous investigations were based on temperature independent [98] and temperature dependent $\varepsilon^{\prime }$ and $b_{110}$, obtained considering a constant thermal expansion [100] of Al${}_{3}$Sc observed experimentally [12] beyond 300 K, and within the Qh approximation [22]. Taking the temperature dependence of the lattice misfit caused by the different thermal expansions of Al and Al${}_{3}$Sc into account, was shown to be crucial [100] to achieve an agreement of the critical size $r_{\mathrm{crit}}$ with experiments. Furthermore, vacancies and Sc solutes were shown to have a similar and opposite effect onto the thermal expansion of Al with a negligible net impact [100]. This validates the applicability of (8), in which these effects are neglected, particularly for the present material system [100]. Figure 7 shows the variation of the critical radius with the temperature including subsequently all finite-temperature contributions to the lattice misfit as well as to the Burgers vector of the matrix. The obtained critical radius is found to increase with temperature, in agreement with the behavior of precipitate-matrix lattice misfit which decreases with temperature (Figure 6).

The critical particle size is a key parameter for the chemo–mechanical coupling between the strain fields caused by the precipitates and the solute kinetics in its vicinity. As recently demonstrated for the case of grain boundary diffusion in an Al-based alloy [101], this coupling leads to nonlinearities in the solute diffusivity. The transition between coherent and incoherent precipitate interfaces explained in this case a minimum in the diffusivity.

As shown in Figure 5, the coherency of particles can be measured by analyzing the atomic strains around them using geometric phase analysis (GPA) images from high resolution electron microscopy. The temperature dependence of the critical size is, however, not readily accessible experimentally, as the annealing experiments are typically performed at fixed temperatures. In fact, as can be seen from Figure 7, all the existing literature data for Al-Sc system lie within 600−800 K with a significantly large scatter of up to ±20–45% in the measured critical size. In the relevant temperature regime, this results in a spread window of over 100 K. Nevertheless, the theoretic predictions fully agree with our experimental data.

The loss of coherency triggers the coarsening rate of the second phase particles. One promising way to improve the coarsening resistance of Al-Sc alloys is to add Zr to it which reduces the lattice misfit of Al${}_{3}$(Sc,Zr) particles with the matrix increasing the critical size at a given temperature, the experimental determination of which is seemingly non-trivial. The ab initio predictions as in Figure 7 can therefore serve as guidelines for the design of technologically relevant advanced structural materials, in which a fine-tuned microstructure is achieved by controlling the desired size of coherent nanoprecipitates using methods such as equal channel angular pressing (ECAP).

## 4. Conclusions

In order to support an investigation of the thermal chemo-mechanical coupling during precipitation in Al-Sc alloys, we have thoroughly studied the difference in thermal expansion between the Al${}_{3}$Sc precipitates and the Al matrix. We have revealed that the thermodynamics is dominated by vibrational and electronic excitations, whereas phonon–phonon anharmonicities and the explicit electron–phonon coupling only yield small corrections. The dedicated dilatometric as well as calorimetric measurements performed in this work underline the high predictive power of the performed calculations throughout the full temperature range that has been considered.

A major focus of the present work was on the correlation of the thermodynamic properties of Al and Al${}_{3}$Sc. To this end, several interpolation schemes between the thermodynamic properties of fcc Al and hcp Sc have been discussed. Vegard’s law for the lattice constants was found to work perfectly for solid solutions, but fails for ordered compounds embedded in the matrix. Similarly, the predicted heat capacity of $L1_{2}$ ordered Al${}_{3}$Sc using the Neumann–Kopp rule was found to differ significantly from the ab initio results and the calorimetric data. Therefore, an alternative approach to model the heat capacity of binaries by using elastic parameters and the Debye model has been suggested, but still needs to be verified for a broader set of binary alloys.

The knowledge of thermal expansions obtained from ab initio modeling allowed us to analyze the elasticity frameworks in the coherency behavior of the particles. It can serve as an input to a phase field modeling of particle growth and microstructure evolution. We have demonstrated that the critical particle size for a coherent/semi-coherent transition has a significant temperature dependence. The good agreement of the simulated values with experimental data underlines the accuracy of the thermal chemo-mechanical coupling considered in the present manuscript.

## Figures and Tables

**Table 1:** Comparison between the calculated (at 0 K) and the experimental values of equilibrium lattice parameter $a_{0}$, bulk modulus $B_{M}$, elastic constants $C_{11}$, $C_{12}$, $C_{44}$, and formation enthalpy $\Delta H_{f}$ of Al${}_{3}$Sc. The error bars for the experimental values (±, wherever available) of elastic constants are mentioned in the last row below the corresponding property. The dagger symbols † and ‡ for experimental values correspond to the data for single-crystal and polycrystalline Al${}_{3}$Sc in Ref. [58], where the Sc concentration was slightly above 25%.

	$a_{0}$	*B*	$C_{11}$	$C_{12}$	$C_{44}$	$\Delta H_{f}$
	(Å)	(GPa)				(eV/atom)
Calc. (Present)	4.103	89.55	184.59	42.03	73.23	−0.445
Calc. (FLAPW) ${}^{\left(a,g\right)}$	4.04 ${}^{\left(a\right)}$	92 ${}^{\left(a\right)}$, 96 ${}^{\left(g\right)}$	189 ${}^{\left(a\right)}$	43 ${}^{\left(a\right)}$	66 ${}^{\left(a\right)}$	−0.5 ${}^{\left(a\right)}$, −0.48 ${}^{\left(g\right)}$
Calc. ( GGA) ${}^{\left(b\right)}$	4.103	91.8	188	43.7	71.4	−0.453
Calc. ( USPP ${}^{\left(e\right)}$/NCPP ${}^{\left(f\right)}$)	4.038 ${}^{\left(e\right)}$	92${}^{\left(e\right)}$	191 ${}^{\left(e\right)}$	43 ${}^{\left(e\right)}$	82 ${}^{\left(e\right)}$	−0.523 ${}^{\left(f\right)}$
Experiments	4.103 ${}^{\left(g\right)}$	–	189 ${}^{(c),†}$	43 ${}^{(c),†}$	66 ${}^{(c),†}$	−0.451 ${}^{\left(d\right)}$
	–	91.5 ${}^{(c),‡}$	182.6 ${}^{(c),‡}$	45.9 ${}^{(c),‡}$	68.4 ${}^{(c),‡}$	–
	–	1.1 ${}^{(c),‡}$	2.1 ${}^{(c),‡}$	0.6 ${}^{(c),‡}$	0.8 ${}^{(c),‡}$	–

^(a)^ Reference [60], ^(b)^ Ref. [61], ^(c)^ Ref. [58] @ 298 K, ^(d)^ Ref. [59] @ 300 K ^(e)^ Ref. [62], ^(f)^ Ref. [14], ^(g)^ Ref. [10].

## Data Availability

The data presented in this study are available on request from the corresponding author. The raw data are currently not publicly available due to huge storage requirements for the molecular dynamics simulations, but could be provided via the pyiron repository https://github.com/pyiron/.
